# From the TPR pathway and the neuroimmune unit to the cutaneous HPA axis: a neuro-endo-immune cross-talk in rosacea

**DOI:** 10.3389/fmed.2026.1800590

**Published:** 2026-07-27

**Authors:** Zijie He, Yan Yan Feng, Tao Ning, Peixuan Wu, Chun Yang, Qian Zhang

**Affiliations:** West China School of Medicine, Sichuan University, Sichuan University Affiliated Chengdu Second People’s Hospital, Chengdu Second People’s Hospital, Chengdu, China

**Keywords:** medical, neuroimmune cell unit, rosacea, skin HPA axis, TRP pathway

## Abstract

The pathogenesis of rosacea is complex, and most of the pathogenesis is not completely clear, but the nervous system, the internal system and the immune system dysregulation is an important factor mediating the pathogenesis of rosacea, yet the three are not a single system’s role, it is the mutual influence, the interaction together mediate the pathogenesis of rosacea. The skin is essentially a whole of neural, internal and immune functions. From the neurological aspect, the skin realizes information transmission by means of neural pathways and receptors, and releases neurotrophins and neuropeptides to influence the organism; from the internal level, the skin not only receives multiple signals, but also has an active part, and possesses a local regulatory function similar to that of the hypothalamus-pituitary–adrenal axis; with regard to the immune function, the skin not only relies on physical barriers, but also on immune cells and related molecules to play a defense function, and also participates in the inflammatory response. In terms of immunity, the skin not only relies on the physical barrier, but also on immune cells and related molecules for defense, and also participates in inflammatory reactions. As a multifunctional organ, the skin maintains homeostasis by regulating peripheral neurologic-endoimmune activities, and at the same time receives bi-directional regulation from the central nervous system, the integumentary system and the immune system. This article describes the three systems that work together in the pathogenesis of rosacea, detailing them in three perspectives: the TPR pathway, the neuroimmune cellular unit, and the cutaneous HPA axis. This article provides a certain summary of the pathogenesis of rosacea, as well as reflections on the underlying mechanisms.

## 1 Introduction

Rosacea as a chronic inflammatory disease of the skin, his clinical presentation is dominated by several manifestations, characterized by paroxysmal, sexual erythema, papules and pustules, capillary dilatation (1). It also includes secondary features such as burning or stinging, plaques, dry appearance, edema, ocular manifestations, perinasal and perioral, and hyperplasia. His typology papulopustular rosacea (PPR): sexual midface erythema with transient midface papules or pustules, or both. Erythematous capillarized rosacea (ETR): sexual erythema of the face with visible capillarization. Pseudorosebaceous Rosacea (PhR): thickening of the skin with irregular nodules and enlargements on the surface, which may occur on the nose, chin, forehead, cheeks, or ears. Ocular Rosacea: foreign body sensation, or tingling sensation, dryness, itching, ocular photosensitivity, blurred vision, sclera or eyes, capillary dilatation or periorbital edema elsewhere, variant granulomatous type:non-inflammatory, firm brown, yellow, or red papules, or uniformly sized nodules (2).

The most recent epidemiologic statistics show that the prevalence of rosacea ranges from 0.09 to 22% in the population, with population studies in Europe and North America yielding higher prevalence rates, with 5.46% of the adult general population suffering from rosacea (3). Three other demographic data suggests that middle-aged females are more prone to rosacea although some scholars have argued that this may be due to the fact that female patients are more inclined to go to the clinic because of this difference in consultation rates yielded this result. More consistently, rosacea has been described as a disease of adults, most prominently in the 45–60 years age range, with almost all cases occurring after the age of 30 (2, 4).

Recent studies have shown that systems biology and metabolomics approaches have provided new perspectives to unravel the complex mechanisms of rosacea. For example, studies of certain natural products (e.g., hydroxyflavanoid A) promoting neurogenesis and axonal regeneration after traumatic brain injury suggest that neurorestorative strategies may be potentially valuable for neurogenic inflammation in rosacea (5). In addition, the synergistic effects of angiogenesis-related factors (e.g., VEGF versus SDF-1α) in vascular myocytes and endothelial precursor cells provide clues to understanding the mechanisms of vasodilation and remodeling in rosacea (6). Neurogenic inflammation and dysregulation of the endosystem and immune system are key factors in the pathogenesis of rosacea. Cutaneous nerves and resident immune cells together form a cutaneous neuro-immune-endoregulatory network, and this synergistic mechanism is critical in maintaining skin homeostasis (3).

## 2 Transient receptor channels (TRP)

Transient receptor channels (TRP) play a major role in the pathogenesis of rosacea as the earliest molecular switches that sense skin exposure and thus cause subsequent changes. In all rosacea subtypes, transient receptor potential (TRP) ion channels have an increased density of distribution in sensory neurons, blood vessels, and immune cells (7). These channels are capable of sensing harmful chemical and physical exposure of the skin, which in turn triggers signals of discomfort, such as pain and itching, and serves as an early warning of potential risks to the body. The TRPV (Transient Receptor Potential Vanilloid Subfamily) receptors are an important subgroup of the TRP cation The TRPV (transient receptor potential vanilloid subfamily) receptors are an important subgroup of the TRP cation channel family, whose members are functionally diverse. This subfamily consists of four non-selective cation channels (TRPV1, TRPV2, TRPV3, TRPV4) and two channels (TRPV5 and TRPV6) that are highly selective for calcium ions (Ca^2+^) (8). TRPV1 is expressed predominantly in neuronal cells and is involved in pain perception and neurogenic inflammatory processes. It is also present in non-neuronal cells such as keratinocytes, which can be activated by capsaicin, high temperatures (>42 °C) and inflammation. TRPV2 is distributed in neuropeptide-containing C-fibers as well as in a variety of non-neuronal cells, such as macrophages and keratinocytes, where it plays a role in innate immunity, inflammation, nociceptive transmission, and hyperthermia sensing. TRPV3 is present in neural tissues, the skin, and blood vessels. It is activated by moderate heat (32–39 °C) and is involved in temperature sensation and regulation of keratinocyte differentiation in the skin. TRPV4 is widely expressed in neuronal and non-neuronal cells such as keratinocytes and endothelial cells. Its activation factors include moderate temperatures (24–34 °C), hypoosmotic-induced cell swelling, and certain inflammatory metabolites. This receptor may act as an osmotic pressure receptor, is involved in vasodilation, and can induce mechanical and inflammatory nociceptive hypersensitivity (8). Further studies have investigated the differential distribution of TPRV subpopulations in different rosacea phenotypes by immunohistochemistry and dual immunofluorescence techniques, with significant immunolabeling of dermal TRPV2 and TRPV3 as well as gene expression of TRPV1 in erythematous capillarized rosacea (ETR). Papulopustular rosacea (PPR) showed enhanced immunoreactivity for TRPV2, TRPV4, and TRPV2 gene expression. In the skin affected by pustular rosacea (PhR), dermal immunostaining for TRPV3 and TRPV4 as well as enhanced gene expression of TRPV1 and TRPV3 were observed in contrast to epidermal TRPV2 staining (8). In other words: TRPV2 is more distributed in ETR and PPR; TRPV3 is more distributed in ETR and PhR; while TRPV4 is more abundant in PPR and PhR (8). In contrast, TRPA (Transient Receptor Potential Anchor Protein Receptor) does not have as many subgroups in mammals, and as far as the current study is concerned, only TRPA1 channels, which are responsible for the integration of a wide range of noxious chemicals and pathologies, have been found in large numbers in humans, and it is a key channel in neurogenic inflammation (9). For a table of the above mentioned TRP channels see Table 1. Specifically, skin perception of inflammation, pain, toxins, and in some cases itch, is mainly transmitted by sensory nerve fibers expressing TRPV (Transient Receptor Potential Vanilloid Receptor) and TRPA1 (Transient Receptor Potential Anchor Protein Receptor 1) cationic channels, and in healthy populations, TRPV in nerve endings usually causes only transient vasodilatation, and quickly returns to normal. However, in the skin tissues of rosacea patients, the expression of TRPV and TRPA1 leads to a rise in Ca^2^ + flux within the synapses of sensory nerve endings and the release of neuropeptides (10, 11). These neuropeptides, in turn, activate the immune cells, such as keratinocytes, that are located around the nerve endings, which triggers neuroinflammatory responses such as facial and edema, and may induce apoptosis of the epithelial cells, impairing the function of the epidermal barrier, and This may induce apoptosis of epithelial cells, impairing the epidermal barrier function and ultimately contributing to the development of skin diseases such as rosacea and atopic dermatitis (AD) (11).

**Table 1:** Data source literature (8).

TRP subtype	ETR	PPR	PhR	Predominant expression cell type	Major activator
TRPV1	Major activator	Major activator	Low quantity	Sensory neurons, keratinocytes	Capsaicin, >42 °C, inflammation
TRPV2	Higher quantity	Higher quantity	Major activator	Macrophages, C-fibers, keratinocytes	High heat, mechanical
TRPV3	Higher quantity	Major activator	Higher quantity	Nerve tissue, keratinocytes, blood vessels	32–39 °C
TRPV4	Major activator	Higher quantity	Higher quantity	Keratinocytes, endothelial cells, neurons	24–34 °C, hypotonic, inflammatory metabolites
TRPA1	Higher quantity	Higher quantity	Major activator	Sensory nerve endings, keratinocytes	Mustard oil, low temperature, inflammatory factor

**Figure 1:**
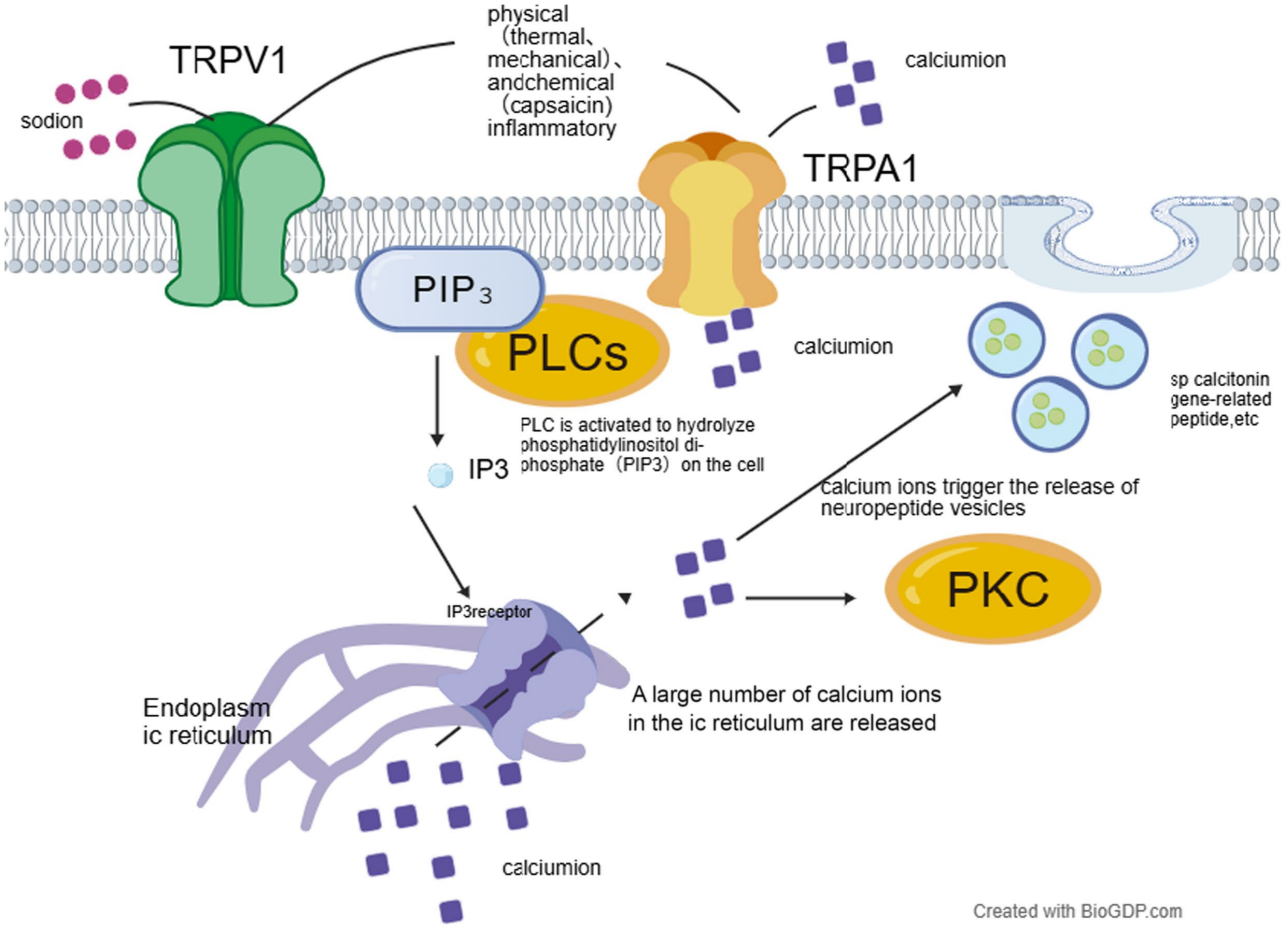
The amplification process of “external calcium influx → activation of PLC → IP₃ signal → calcium replenishment in the endoplasmic reticulum” is used to amplify pain or heat sensation signals.

Activation of TRP in sensory nerve endings leads to depolarization of the cell membrane by cations (Na^+^, Ca^2+^), and the molecular signaling mechanism: The cascade of reactions from TRP activation to neuropeptide release can be summarized as follows: (Thermal, chemical, mechanical) → Opening of TRPV1/TRPA1 channels → Ca^2+^ inward flow → Activation of phospholipase C (PLC) → hydrolysis of PIP_2_ to generate IP_3_ → IP_3_-induced Ca^2+^ release from the endoplasmic reticulum → activation of protein kinase C (PKC) → vesicular translocation of neuropeptides (SP, CGRP) to presynaptic membranes for release → action on effector cells, such as cells. This signaling pathway is aberrantly amplified in rosacea by TRP expression (see Figure 1). The abnormal number of TRP receptor pathways in patients with rosacea results in frequent action potentials in the axon initiation segment, and ultimately by nerve fibers, which transmit action potentials to affect various types of cells within the skin and mediate inflammatory responses.

## 3 Neuroimmune cell units (NICUs)

When the organism is subjected to foreign, the nerve synapse receives, causing an action potential, and when the impulse is transmitted to the nerve endings, TRP channels release neuropeptides, which affect the neuroimmune cell units, then mediate the inflammatory response. In recent years, there has been increasing evidence to support an important role for neurogenic inflammation in the pathogenesis of rosacea. Understanding the nerve fibers and the neurotransmitters they release, as well as the target cell types, can lead to a better understanding of the pathogenesis of rosacea. The TRP channels and neuroimmune cell units (NICUs) can show the process of neurogenic inflammation more clearly and help us better understand the pathogenesis of rosacea. The skin is mainly innervated by sensory nerves, which are classified according to their diameter, degree of myelination, and conduction velocity into A-*β*, A-*δ*, and C fibers (12). The A-β fibers, which are highly myelinated and have fast conduction velocities, innervate specialized sensory end-organs, such as Meissner’s vesicles, Pacini’s vesicles, Merkel cells, and Ruffini’s vesicles (13, 14) also called sensory vesicles. Functionally, different sensory vesicles respond to different types, for example, Meissner’s vesicles are innervated by two mechanoreceptor subtypes that show different responses to different tactile sensations, whereas Ruffini’s vesicles are considered temperature receptors (14), and Pacini’s vesicles and Merkel’s cells respond to pressure and vibration (15). Structurally, these vesicles consist of sensory axon terminals and peripheral glial cells (14). The presence of these vesicles, then, better assists the skin in sensing foreign. a-*δ* fibers are relatively poorly myelinated, conduct slowly, and respond to mechanical and thermal nociception as well as non-injurious sensations of heat and cold. c fibers, on the other hand, are unmyelinated, have the slowest conduction, and can be activated by heat, chemical, and mechanical activation (16–18). These nerve fibers are surrounded by a distribution of immune cells such as cells, dendritic cells, macrophages, intrinsic lymphocytes and γδ T cells, which together constitute neuroimmune cell units (NICUs) (19), which synergistically maintain skin homeostasis (19). Both the nervous and immune systems of the skin perform “sensory” functions: immune cells recognize pathogens and sensory nerves detect harmful ones. Both collaborate to respond to changes. Once detected (especially injurious), the cutaneous nervous system usually communicates with the immune system through the release of neurotrophic factors (NTs) and neuropeptides (NPs), which triggers a series of subsequent responses known as “neuroimmune interactions” (19). For example, when peripheral sensory nerves are damaged or signaled by inflammation, the peripheral nerves of the skin release a variety of neuropeptides and neurotransmitters, such as substance P (SP), calcitonin gene-related peptide (CGRP), vasoactive intestinal peptide (VIP), and pituitary adenylyl cyclase-activating peptide (PACAP) (20), which activate the MCs and induce degranulation to release histamine, tryptase, interleukins, and other neurotransmitters (21). Trypsin, interleukins and several pro-inflammatory cytokines, which trigger pro-inflammatory effects, abnormal vascular proliferation and inflammatory cells (21, 22). These mediators have a positive regulatory effect on nerve fibers, which further release neuropeptides, thus forming a bidirectional positive feedback loop with MCs, continuously amplifying the inflammatory cascade response and driving disease progression (7). For example, trypsin-like enzymes bind to protease-activated receptor 2 (PAR-2) on sensory nerves, enhancing injury perception and promoting neuropeptide release, exacerbating the local inflammatory response; and MCs-derived IL-31 and IL-13 initiate the process of sensory neuron growth and outgrowth (23). The interaction of these neuropeptides with sensory neurons further extends the inflammation. In summary, the aberrant expression of the number of TRP channels is the first step in neurogenic inflammation in rosacea, whereas the neuroimmune cellular unit is the subsequent response of the organism to the first step, and these two components are indispensable in neurogenic inflammation. In short, the process of neurogenic inflammation, as described above, occurs when the organism receives mechanical or temperature or chemical from the outside world → Abnormal activation of TRP channels (V1/V3/V4/A1) (switching) → Excitation of sensory nerve endings and release of neuropeptides → Activation of cells in the NICUs (effectors) → Cellular release of inflammatory mediators → Appearance/edema/pruritus (clinical manifestations) → Inflammatory mediators reversed to the Nerve endings → Nerves release more neuropeptides (positive feedback) → Inflammation amplifies and amplifies disease progression.

**Figure 2:**
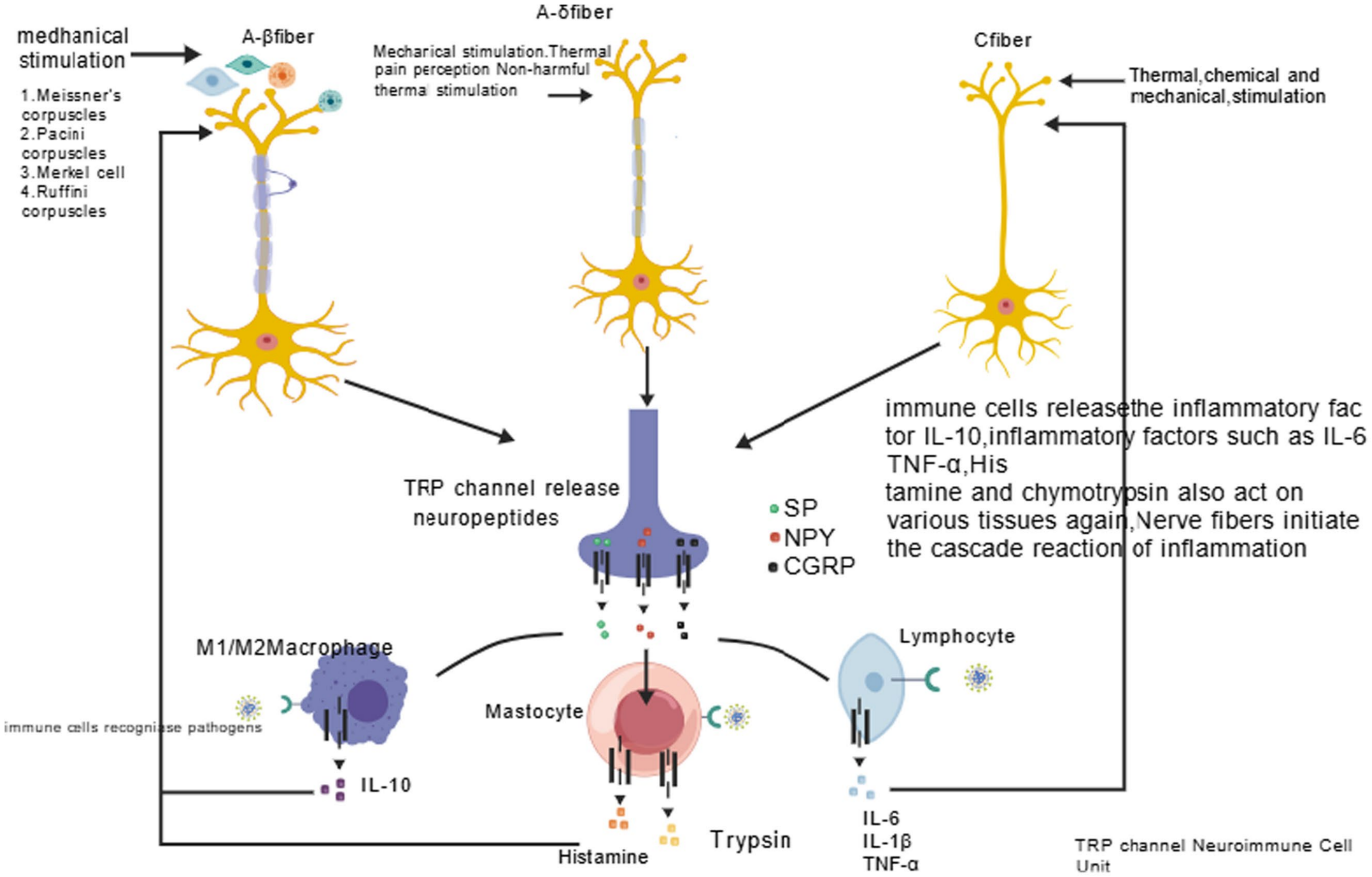
External stimulus → Nerve releases signal → Immune cells respond → Release inflammatory factors → This in turn activates the nerve, forming a positive feedback loop, which is involved in pain, allergy and inflammation processes.

The course of action of specific neuroimmune cell units can be seen in Figure 2.

### 3.1 Skin HPA axis

Activation of the HPA axis begins with the production of corticotropin release (CRH) in the hypothalamus, followed by activation of the CRH receptor type 1 (CRHR1) in the anterior pituitary gland and induces the cleavage of opioid opiomelanocortin progenitors (POMCs) into corticotropin (ACTH), *α*-melanotropic cells (α-MSHs), and *β*-endorphins (β-ENDs) (24). ACTH adrenal cortical glycocortex (GCs), GCs respond to stressors by suppressing the immune response, reducing inflammation in response to stress and inhibiting the HPA axis through negative feedback (24). When the body is in an inflammatory state, the anti-inflammatory effect of cortisol decreases significantly, and according to the current study, we hypothesize that this may be related to: (1) Diminished receptor activity, (2) limited number of receptors, resulting in a limited anti-inflammatory effect, and (3) chronic inflammation, which activates certain pathways and directly inhibits receptor function (25). Decreased anti-inflammatory capacity is also a factor in the chronic inflammation of the face in patients with rosacea. When a stressor acts on the skin, keratin-forming cells (KCs) in the skin produce class products, similar to those produced during systemic stress events, such as CRH, POMC, *β*-END, ACTH, and *α*-MSH (26). In addition, corticosteroid synthases such as CYP11A1, 3β-HS CPY17A1, CYP21A2, and CYP11B1 are expressed in keratinocytes (KCs), resulting in the production of corticosterone and cortisol (27, 28), which further demonstrates the presence of the skin HPA axis. The skin also expresses a variety of receptors, such as the glucocortex (GCs) and mineralocortex (MCs), which intervene in epidermal development and homeostasis through glucocortical receptors (GR) and mineralocortical receptors (MR), which are important for the skin to maintain homeostasis. All kinds bring their biological effects into the skin by binding to receptors in the skin. Therefore, when patients with rosacea are physically, chemically, and biologically harmful, skin homeostasis will also be affected (29). The functional tissue of the skin HPA axis, which is composed of CRH and POMC signals, operates in peripheral organs such as the skin (30). Interacts with the central HPA axis and acts as a mutual pathway, co-mediating the occurrence of facial inflammation in patients with rosacea and. In the following, this review will describe the key components of the skin HPA axis and the known pathogenic processes, respectively.

#### 3.1.1 POMC signal

It is widely recognized that pro-melanocortin (POMC) signaling plays a key role in maintaining body homeostasis. POMC is not only expressed in hypothalamus pituitary and other brain regions but also in immune cells and a variety of non-neuronal peripheral organs. In the skin, almost all cell types, including keratinocytes, melanocytes and fibroblasts, express POMC precursor proteins. This precursor is specifically processed in different and cell types to produce a series of bioactive peptides, such as corticotropic (ACTH) and its truncated forms (ACTH1-13) and (ACTH1-17), melanocytosin (*α*-MSH, *β*-MSH and *γ* principal-4.3-MSH), β-lipoprotein and β-endorphin, etc., and to the extracellular (30). These peptides can promote epidermal melanogenesis, enhance skin barrier function, exert cytoprotective effects, regulate hair growth, and participate in the regulation of lipogenesis, sebum and eccrine sweat gland function (30). In addition, they also have a variety of physiological effects such as immunosuppression, antibacterial, anti-fibrosis, radiation protection, and thermoregulation. They can participate in pain regulation by acting on corresponding receptors in nerve endings, and can affect central energy to control the body (30). Among them, the anti-inflammatory, immunosuppressive and antipyretic functions of *α*-MSH, the precursor of ACTH, are particularly clear in the skin and immune system (30). At present, the mechanism of POMC signal acting directly on rosacea is still unclear, and it is more likely to regulate ACTH precursor, which acts together with CRH to produce cortisol and affect the body.

#### 3.1.2 CRH

Skin stressors such as ultraviolet radiation (mainly UVB), microbial antigens, and physical trauma (including damage) can produce CRH directly from skin-resident cells and immune cells, or indirectly be subjected to pro-inflammatory cytokines induced by CRH and others (30). They regulate homeostasis mainly by activating CRH receptor type 1 and CRH receptor type 2. CRH-R1 is predominantly found in different regions of the pituitary gland and brain, while CHR-R2 is found in the heart, skeletal muscle, muscle and immune cells (31). As the central coordinator of the central HPA axis and the skin HPA axis, the main role of CRH in the center is pro-inflammatory, which is achieved by acting on the production or release of CRH-R1POMC peptide, and the cortisol production and release of ACTH, as well as the activation of the central hypothalamic–pituitary–adrenal (HPA) axis. The released glucocortex acts on systemic cells and mediates anti-inflammatory effects. And CRH is mainly pro-inflammatory in the periphery, (in the periphery, CRH will also produce cortisol together with POMC peptide and ACTH produced by keratinocytes (27, 28), but due to the small number of CRH-R1, the peripheral anti-inflammatory ability is weak). CRH acts on the CRH-R2 of immune cells in the periphery, which can directly cause cell degranulation and release histamine, has significant vascular permeability, and regulates the production of IL-18 in keratinocytes (KCs) and IL-6 and IL-8 in sebum cells. These will mediate the increase of MAP kinase (MAPK) and the enhancement of NF-kB pathway (both are important pathways for regulating pro-inflammatory factors), and lead to inflammatory cell aggregation and facial erythema (32). CRH also acts on CRH-R2 on the vascular muscles, making the vascular muscles relaxing and blood vessels dilating, which is also one of the important reasons for facial telangiectasia and facial erythema in patients with rosacea (31). In addition, CRH also increases the number of TRPV1 (transient receptor potential vanillin receptor 1) and TRPA1 (transient receptor potential anchor protein receptor 1), and strengthens the expression of cation channels in nerve endings innervated by CRH (30).

#### 3.1.3 The process of specific central and skin HPA axis action

**Figure 3:**
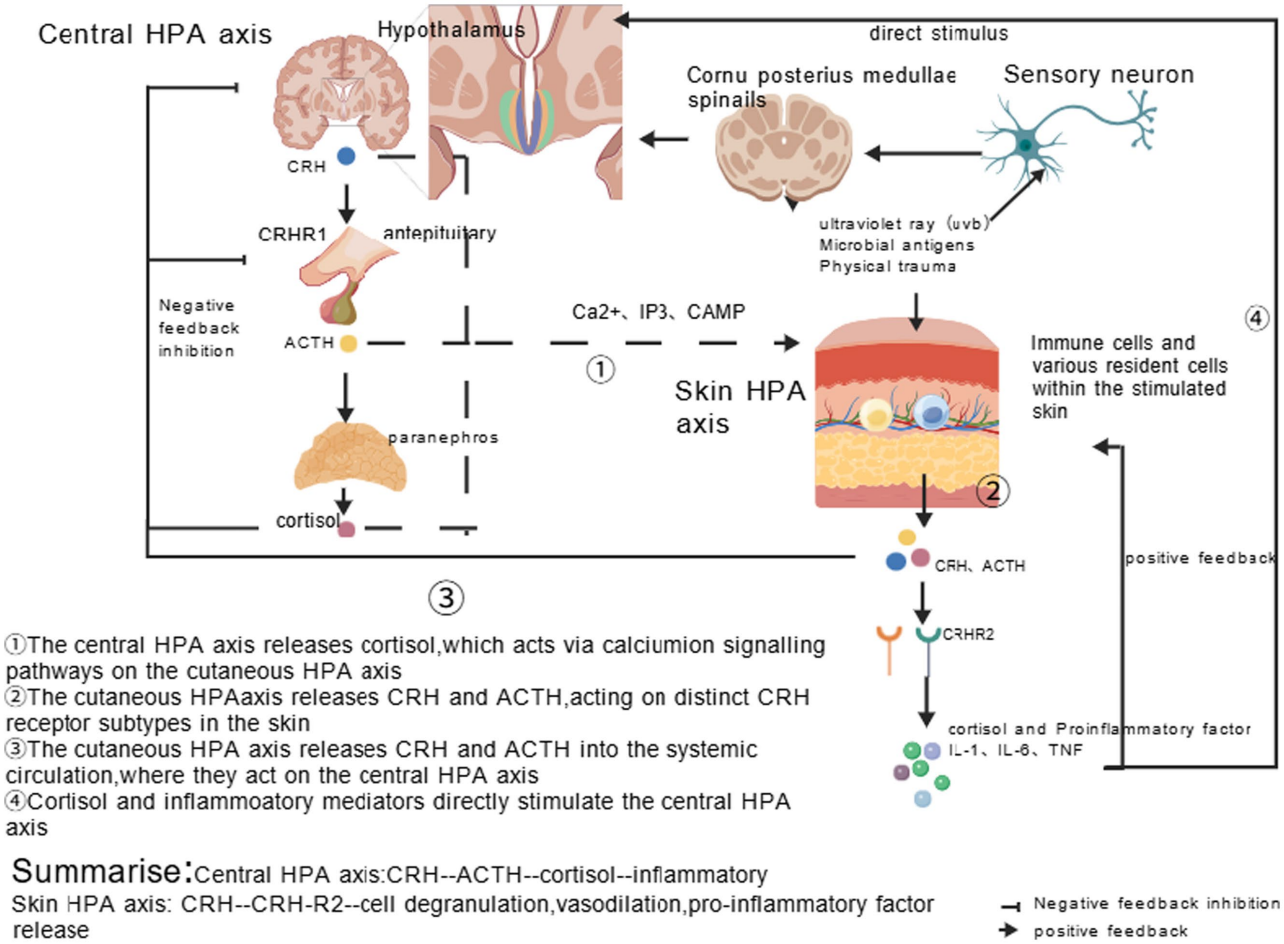
The central HPA axis secretes cortisol to exert anti-inflammatory effects, while the local HPA axis promotes inflammation. These two systems interact with each other through hormones and cytokines, forming a stress-inflammation network both at the systemic and local levels.

The specific pathogenic mechanisms of the central and cutaneous HPA axes are as follows. Rosacea begins with the perception of external physics, chemistry and biology by the cutaneous nervous and immune systems. When patients are stressed due to mechanical, temperature, chemical or emotional stress, the corticotropic release (CRH) released by the hypothalamus is transported to the anterior pituitary gland, activates the CRHR1a receptor on it, and induces the cleavage of opioid melanocorticogen (POMC) to corticotropic (ACTH) melanocytosin (*α*-MSH), *β*-endorphin release (33). Subsequently, ACTH acts on the adrenal cortex by activating cortisol such as melanocortin type 2 receptor (MC2) and glucocortex. Cortisol suppresses immune responses, reduces inflammation, and inhibits CRH production in the hypothalamus versus ACTH production in the pituitary through negative feedback mechanisms in response to stress states (26, 34). At the same time, CRH, ACTH, and cortisol in the central pivot can also couple cAMP, IP3, or calcium signaling pathways, and act on peripheral skin tissues through para-, autonomic, neurological, and intraperitoneal (35–37), mediating pro-inflammatory effects in peripheral skin tissues. Both skin keratinocytes (KCs) and fibroblasts synthesize CRH, ACTH when the skin is subjected to external physical, chemical and biological, or systemic psychological and physiological stress (38). CRH and its ACTH-related peptides not only cause cell degranulation, vascular permeability, and exert pro-inflammatory effects, but also can trigger pro-inflammatory responses by releasing inflammatory factors (IL-1, IL-6, tumor necrosis factor TNF) through CRH receptor type 2 (CRH-R2) (24, 39, 40). On the other hand, pro-inflammatory cytokines such as IL-1, IL-6, and tumor necrosis factor (TNF) not only release CRH and ACTH directly from the hypothalamus and pituitary (41, 42), but also form positive feedback with cells such as keratinocytes, amplifying inflammatory effects. The process of CRH production by skin keratinocytes and fibroblasts can be directly activated by ultraviolet rays (mainly UVB), microbial antigens, physical trauma, etc., and can also be indirectly activated by pro-inflammatory cytokines induced by CRH or other factors (43). It is worth noting that UVB and other factors can regulate the expression and composition of CRHR subtypes (43, 44). Therefore, patients with rosacea may in some cases, because their skin CRH-R2 receptors are more expressed than CRH-R1, resulting in a pro-inflammatory response than an anti-inflammatory response in the peripheral skin (45). To sum up, when patients with rosacea are under stress, the anti-inflammatory system of the body takes the lead in responding to the anti-inflammatory effect of the glucocortex. The mechanism of the decline in the anti-inflammatory effect of the glucocortex (GC) under chronic inflammation may include: (1) GR*α* receptor phosphorylation leads to nuclear translocation disorder; (2) The expression of GR antagonist GR *β* increased; (3) Inflammatory factors (such as TNF-α, IL-1) activate AP-1 and NF-κB, competing with GR for transcriptional co-activators; (4) Abnormal metabolism of cortisol precursor. Together, these mechanisms contribute to the presence of facial inflammation in patients with rosacea (45). However, under the effect of peripheral pro-inflammatory factors, the inflammation of the body weakens the anti-inflammatory ability of the body (the specific weakening is not yet clear) (25), and the inflammation of the body cannot be effectively inhibited, while the pro-inflammatory factors in the body Continuously, the decline of anti-inflammatory ability and the strengthening of pro-inflammatory ability lead to the chronic inflammation of the body. The above HPA axis process can be seen in detail in Figure 3.

Emotional stress activates the HPA axis, the release of inflammatory factors that activate inflammatory pathways and impair skin barrier function (46). In a survey conducted by the American Rosacea Association (NRS), emotional stress was reported as one of the most common rosacea triggers, affecting 79% of respondents (47). In another similar survey by the NRS, 69% of rosacea patients reported that emotional stress was associated with increased acne each month (47). Related reports that 67% of patients can aggravate rosacea by focusing on reducing stress alone (47). First-line management of rosacea includes avoiding stressors, such as sun exposure, and taking adequate sun protection and moisturizing measures. Meditation and yoga are thought to act on the HPA axis, thereby inflaming factor levels (46), so relieving emotional stress is also a new direction for rosacea treatment.

## 4 Summary

The pathogenesis of rosacea initiates when the body is exposed to external physical, chemical, biological and emotional stimuli. Such stimuli are first received by neural synapses, which trigger the TRP pathway to produce neuropeptides. Acting as effector units, neuroimmune cellular units then release inflammatory mediators. Some of these mediators retrogradely stimulate nerve terminals to drive additional release of inflammatory mediators, leading to progressive inflammatory exacerbation and disease advancement (48). The remaining mediators activate both the central hypothalamic–pituitary–adrenal (HPA) axis and the cutaneous HPA axis, with corticotropin-releasing hormone (CRH) serving as a key mediator. This results in attenuated central anti-inflammatory responses and persistent peripheral pro-inflammatory status, further worsening clinical manifestations. Accumulating CRH additionally upregulates the abundance of TRP pathways and cation channels on nerve terminals, rendering neurogenic inflammation more prone to development and establishing a self-perpetuating vicious inflammatory cycle (49). Accordingly, this pathological cascade is categorized into three sequential phases: (1) Initiation phase (TRP channels): Extrinsic stimuli including temperature fluctuations, chemical irritants, mechanical injury and emotional stress activate cutaneous sensory nerve terminal channels such as TRPV1 and TRPA1, triggering Ca^2+^ influx and subsequent secretion of neuropeptides including substance P (SP) and calcitonin gene-related peptide (CGRP). (2) Amplification phase (Neuroimmune Cellular Units, NICUs): Released neuropeptides activate adjacent mast cells, prompting secretion of inflammatory mediators such as histamine, tryptase and interleukin-31 (IL-31). These mediators induce flushing and edema, and concurrently retro-stimulate nerve terminals to establish a positive feedback loop for inflammatory amplification. (3) Maintenance phase (HPA Axis): Inflammatory mediators activate the local cutaneous HPA axis (CRH → pro-inflammatory cytokines → vasodilation); meanwhile, the central HPA axis exhibits impaired anti-inflammatory capacity via glucocorticoid resistance, facilitating chronic inflammation. CRH further upregulates TRP channel expression, which closes the aforementioned positive feedback loop.

Taken together, rosacea is a disease originating from TRP pathway activation, which sequentially engages neuroimmune cellular units followed by persistent regulatory effects from both central and cutaneous HPA axes throughout disease progression. In conclusion, the interplay between central/cutaneous HPA axes and neuroimmune cellular units redefines rosacea as an illness modulated by psychological stress, social determinants and neuroendocrine-immune crosstalk.

## 5 Outlook

The specific pathogenic mechanism of rosacea involves multiple factors and links, which has not been fully explained by the medical community at present (50). Recent studies have further revealed that its pathogenesis is highly complex. It is believed that there is a close interaction between neurosensory dysfunction and immune regulation mechanisms and internal systems, and the three jointly participate in and promote the occurrence and development of diseases. Blocking the association between these pathogenic pathways is expected to become a new strategy for the treatment of rosacea. Because there are multiple key nodes between these mechanisms, intervention at any of them may alleviate the condition. Therefore, the pathological mechanism of this disease involves a wide range, and the corresponding treatment approaches are also diverse. Existing studies have confirmed that TRP pathway, neuroimmune cell unit and skin HPA axis play a key role in the pathogenesis, but the specific mechanism of action remains to be explored. With the recognition of the combined action of these three aspects, holistic therapeutic approaches can be attempted that combine conventional immunotherapy with interventions to restore intraneural balance (e.g., stress management, CRH-R2 antagonists, TRPV1 antagonists) (50), ultimately improving skin and systemic outcomes. In the future, more clinical trials need to be carried out, especially the application of single-cell multi-omics and spatial transcriptomics technologies, to further reveal the molecular signaling pathways of rosacea, to find potential therapeutic targets, so as to optimize existing treatment strategies and provide clinical diagnosis and Treatment provides new directions (51).
